# Epicardial adipose tissue volume outperforms density in association with cardiorenal complications in hypertensive patients

**DOI:** 10.1186/s12944-026-02873-x

**Published:** 2026-02-12

**Authors:** Hong-Xing Wu, Mengmeng Liu, Weibin Mao, Sijing Luo, Xiaolan Ouyang, Anni Xiong, Ji Luo, Xiangbo Meng, Yemin Li, Xiangjie Tian, Suhua Li, Man Han, Zhanao Meng, Libao Liu, Jiaojiao Wu, Feng Shi, Xianguan Yu, Xixiang Tang, Jie Qin

**Affiliations:** 1 Division of Cardiovascular Medicine, The Third Affiliated Hospital, Sun Yat-sen University, Guangzhou, China; 2 Department of Radiology, The Third Affiliated Hospital, Sun Yat-sen University, Guangzhou, China; 3 Division of Endocrinology and Metabolic Diseases, The Third Affiliated Hospital, Sun Yat-sen University, Guangzhou, China; 4 State Key Laboratory of Cardiovascular Diseases, Fuwai Hospital, Chinese Academy of Medical Sciences & Peking Union Medical College, Beijing, China; 5 Division of Neurology and Cardiology, The Affiliated Hospital, Xizang Minzu University, Xianyang, China; 6 Division of Cardiothoracic Surgery, The Third Affiliated Hospital, Sun Yat-sen University, Guangzhou, China; 7 Department of Research and Development, United Imaging Intelligence Co., Ltd., Shanghai, China; 8 VIP Healthcare Center, The Third Affiliated Hospital, Sun Yat-sen University, Guangzhou, China; 9 Guangdong Provincial Key Laboratory of Diabetology & Guangzhou Municipal Key Laboratory of Mechanistic and Translational Obesity Research, The Third Affiliated Hospital, Sun Yat-sen University, Guangzhou, China

**Keywords:** Epicardial adipose tissue, Cardiorenal complication, Hypertension, Coronary heart disease, Heart failure, Renal dysfunction

## Abstract

**Background:**

Epicardial adipose tissue (EAT) dysfunction is closely related to a variety of cardiovascular diseases. However, controversy persists regarding the association between EAT characteristics and hypertension (HTN). The present study aims to clarify the changes of EAT volume and density in HTN patients, and explore their relationships with cardiorenal complications.

**Methods:**

A total of 257 individuals were enrolled in this study for analysis, including 156 HTN patients and 101 non-HTN participants. EAT volume and density were measured using coronary computerized tomography angiography (CCTA). Cardiorenal complications were evaluated by laboratory indicators, echocardiography, and CCTA. Correlation analysis was used to examine the relationship between EAT characteristics and various cardiorenal complications. Mediation analysis was performed to test whether EAT characteristics played mediating effects between HTN and cardiorenal complications.

**Results:**

Compared to non-HTN participants, HTN patients exhibited a markedly higher volume of EAT (154.40 ± 53.31 vs. 132.49 ± 45.88 cm^3^, *P* < 0.001). Nevertheless, there was no significant difference in density between the two groups (-80.22 ± 6.84 vs. -79.25 ± 7.16 HU, *P* = 0.279). Correlation analyses showed that the volume of EAT was strongly correlated with most indicators of cardiorenal complications (all *P* < 0.05), including severity of coronary artery disease (plaque volume, CACS, and CT-FFR), abnormal cardiac structure and function (aorta, LA, IVS, LVPW, LVEF, E/A ratio, and NT-proBNP), and renal dysfunction (BUN, serum creatinine, CysC, and eGFR), while the density of EAT was only correlated with partial indicators of cardiorenal complications. Mediation analysis found that EAT volume had significant mediating effects between HTN and cardiac complications (mediation proportions: 68.30% for plaque volume, 40.67% for CACS, 13.51% for CT-FFR, 10.63% for aorta, and 12.78% for IVS, respectively).

**Conclusion:**

EAT volume, rather than density, is significantly increased in HTN patients and closely associated with cardiorenal complications. This finding provides a new perspective for preventing HTN complications by targeting the reduction of heart-specific visceral adipose tissue.

**Graphical Abstract:**

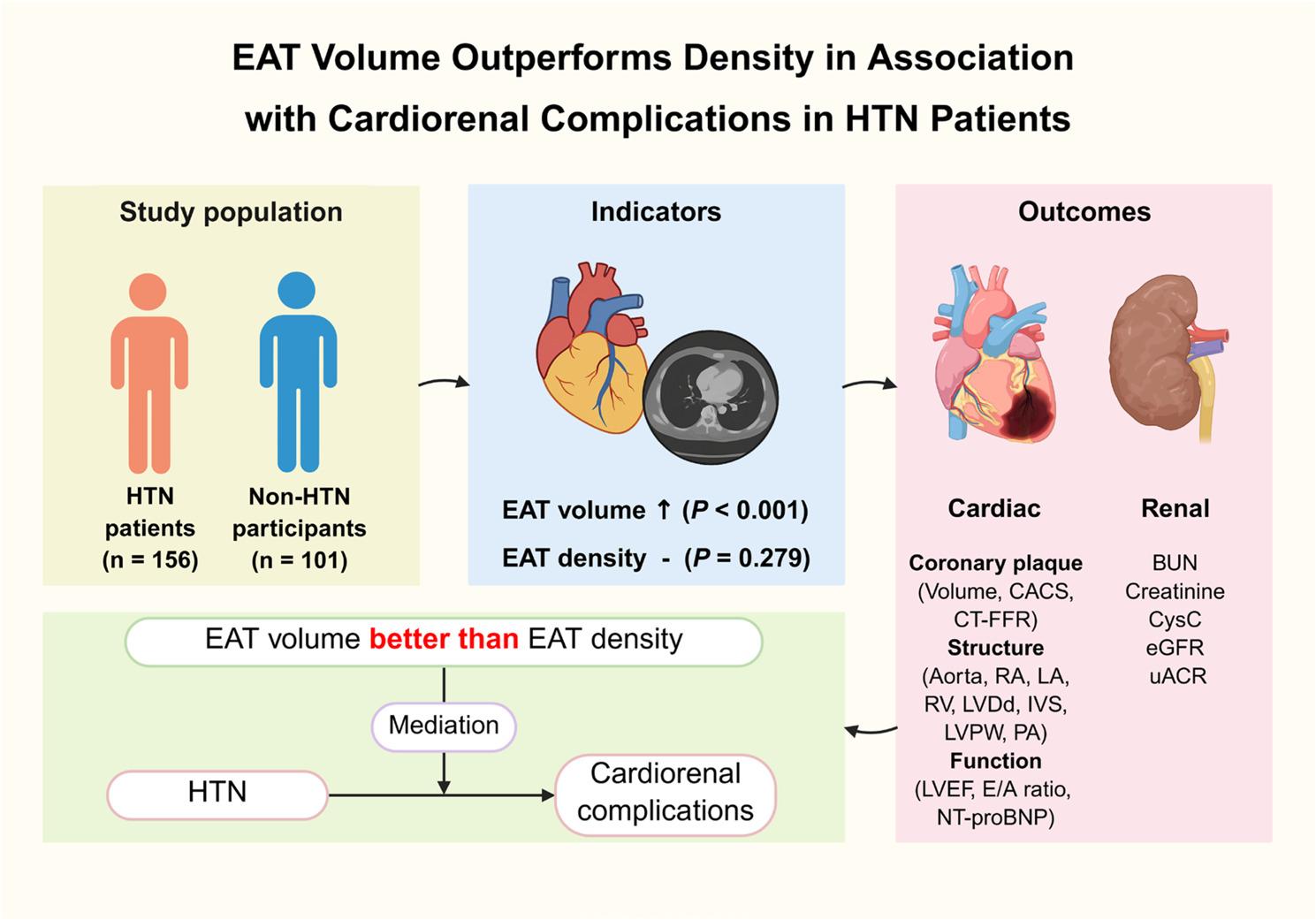

**Supplementary Information:**

The online version contains supplementary material available at 10.1186/s12944-026-02873-x.

## Introduction

Hypertension (HTN) is one of the most common chronic diseases and an important risk factor for cardiovascular diseases worldwide. Its prevalence continues to rise globally and has become a major public health challenge [1, 2]. HTN can lead to a variety of complications in multiple organs throughout the body, causing a large number of deaths annually [3, 4]. In the progression of disease, the heart and kidney are the main organs affected by the long-term burden of HTN, so the incidence of cardiorenal complications is higher than other complications [4, 5]. Therefore, it is necessary to find more appropriate targets for prevention and treatment of HTN and cardiorenal complications.

Epicardial adipose tissue (EAT) is the heart-specific visceral adipose tissue deposited between the visceral layer of the pericardium and the myocardium [6, 7]. In general, EAT has a protective and supportive effect on the heart. However, excessive accumulation or dysfunction of EAT can lead to adverse myocardial remodeling and affect the electrical conduction of cardiomyocytes, through inflammation, oxidative stress, metabolic disorder, and mechanical stress, and further participate in the occurrence and development of cardiovascular diseases [7–9]. Thus, understanding, detecting and intervening the characteristics of EAT are important components of prevention and treatment of cardiovascular diseases. Previous studies have shown that EAT is closely related to cardiovascular diseases, such as coronary artery disease, heart failure, and atrial fibrillation [10–17]. However, the relationship between EAT and HTN, especially with HTN complications, is still controversial, which is an important issue that has not been resolved.

Moreover, the volume and density of EAT exert distinct influences on different diseases. For instance, in COVID-19 pneumonia, the volume of EAT, compared with its density, can better independently predict the risk of mechanical ventilation or death in patients [18]. Conversely, for metabolic syndrome, EAT density demonstrates superior diagnostic efficacy over volume [19]. Regarding cardiovascular diseases, EAT density holds greater priority for evaluating high-risk coronary plaques in coronary artery disease and better guides the assessment of metabolic risk in heart failure with preserved ejection fraction (HFpEF) [12, 19]. However, it remains unclear whether EAT volume or density is more important in HTN patients, which constitutes a significant unresolved question.

To address this issue, a cross-sectional study was carried out to characterize EAT volume and density in HTN patients, and explore their relationships with the cardiorenal complications. This study aims to provide a new perspective for the prevention and treatment of HTN-related cardiorenal complications by targeting the characteristics of heart-specific visceral adipose tissue.

## Methods

### Study population

This was a single-center, cross-sectional study conducted at the Third Affiliated Hospital of Sun Yat-sen University from May 2021 to May 2023. Adult HTN patients and non-HTN participants who were hospitalized in the Division of Cardiovascular Medicine and underwent laboratory tests, echocardiography, and coronary computerized tomography angiography (CCTA) were enrolled. According to the guidelines issued by the European Society of Cardiology (ESC), HTN was defined as systolic blood pressure (SBP) ≥ 140 mmHg and/or and diastolic blood pressure (DBP) ≥ 90 mmHg [20]. Exclusion criteria included: (1) Patients were younger than 18 years old; (2) Patients previously undergone percutaneous coronary intervention (PCI) or coronary artery bypass grafting (CABG); (3) Patients previously suffered from coronary artery disease, heart failure, arrhythmia, and renal dysfunction before being diagnosed with HTN; (4) Patients with severe organic heart diseases such as valvular heart disease, hyperthyroidism-induced cardiomyopathy, dilated cardiomyopathy, and hypertrophic cardiomyopathy; (5) Patients with severe or acute hepatic insufficiency due to various etiologies; (6) Patients with hyperthyroidism, hysterectomy, adrenalectomy and other endocrine hormone disorders; (7) Patients with rheumatic immune diseases such as Sjogren’s syndrome and systemic lupus erythematosus; (8) Patients suffered from advanced cancer; (9) Patients with infectious diseases such as syphilis, leptospirosis, viral hepatitis; (10) Female patients who were pregnant or lactating; (11) Patients with incomplete information. Finally, the remaining patients were enrolled for analysis. This study had been approved by the Ethics Committee of the Third Affiliated Hospital, Sun Yat-sen University and complied with the ethical principles of the Declaration of Helsinki [21]. In addition, all enrolled patients provided written informed consent.

### Data collection

Demographic and clinical characteristics, including age, sex, body mass index, blood pressure (SBP and DBP), heart rate, adverse life behaviors (smoking and drinking), past medical history and medications history, were collected from all included patients through the electronic medical record system of inpatients. After the overnight fasting for 8 h, venous blood samples were drawn from all enrolled patients, and biochemical indicators were detected by Hitachi 7180 automatic biochemical analyzer (Hitachi, Ltd., Tokyo, Japan). Biochemical indicators include fasting blood glucose (FBG), total cholesterol (TC), triglyceride (TG), high-density lipoprotein cholesterol (HDL-C), low-density lipoprotein cholesterol (LDL-C), lipoprotein a, N-terminal pro-B-type natriuretic peptide (NT-proBNP), uric acid, blood urea nitrogen (BUN), serum creatinine and cystatin C (CysC). In addition, glycosylated hemoglobin A1c was measured by D-10 hemoglobin testing program with high-performance liquid chromatography (Bio-Rad Laboratories, Inc., California, USA), and estimated glomerular filtration rate (eGFR) was calculated by using the chronic kidney disease epidemiology collaboration (CKD-EPI) CysC equation [22]. Besides, urinary albumin was measured using turbidimetric immunoassay in three consecutive urine collections and expressed as urinary albumin-to-creatinine ratio (uACR).

### Measurement of EAT and coronary plaque

**Fig. 1:**
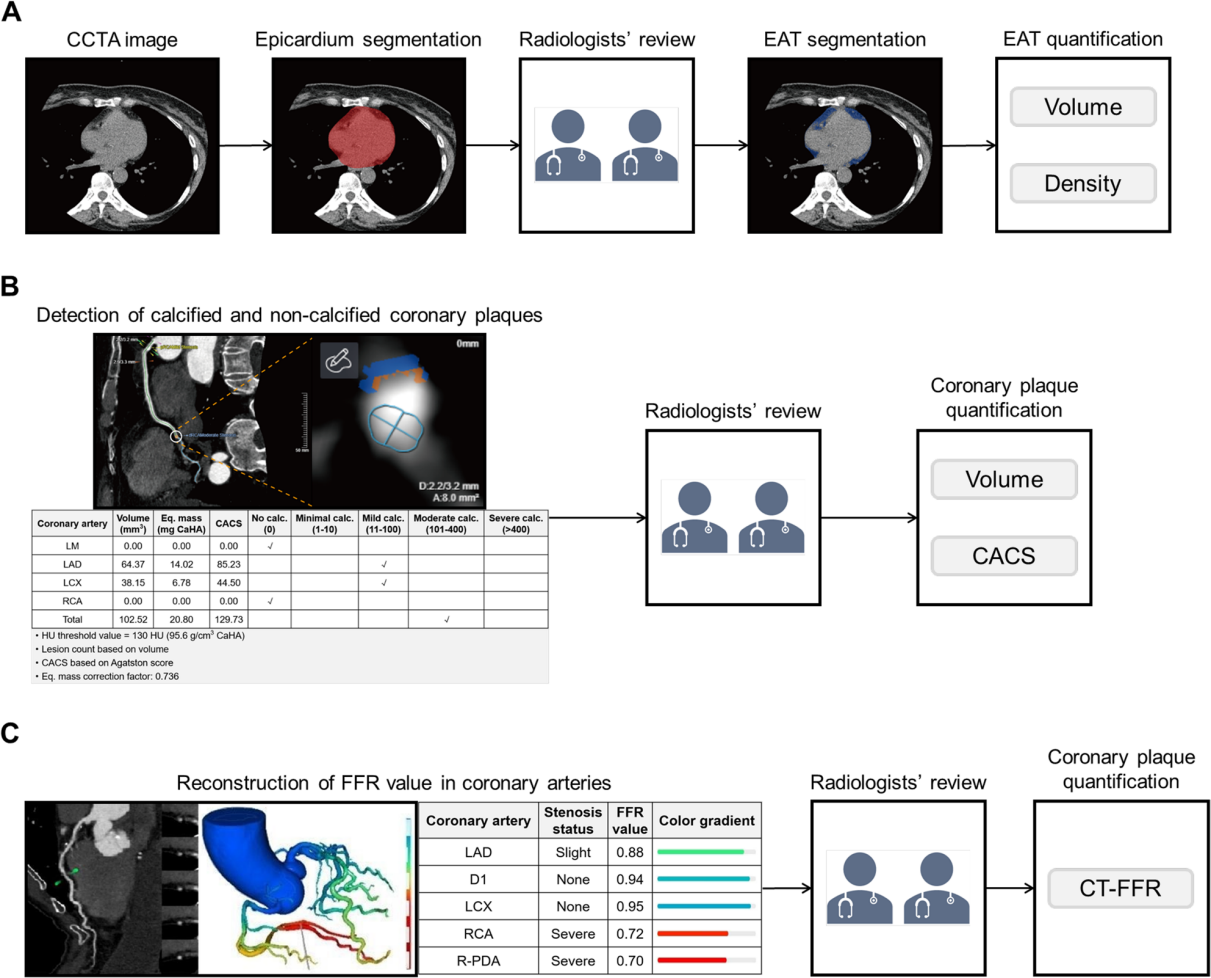
Measurement of EAT and coronary plaque. Methods and procedures for measuring EAT (**A**) and coronary plaque (**B** and **C**) characteristics by using the uAI Research Portal. EAT, epicardial adipose tissue; CCTA, coronary computerized tomography angiography; LM, left main coronary artery; LAD, left anterior descending; LCX, left circumflex; RCA, right coronary artery; CACS, coronary artery calcification score; FFR, fractional flow reserve; ‌R-PDA, right posterior descending artery. CT-FFR, computerized tomography-fractional flow reserve

As shown in Fig. 1, the processes of automatic segmentation and accurate quantification of EAT and coronary plaque were conducted via the uAI Research Portal (uRP, United Imaging Intelligence Co., Ltd., Shanghai, China) [23], which is specifically designed for clinical scientific research and incorporates a wealth of artificial intelligence (AI) algorithms. Detailed measurement methods are provided in the Supplementary Methods.

In the quantification of EAT, a three-step approach was implemented. Initially, the epicardium was automatically segmented using a deep learning model (nnUNet) on CCTA images, and then it was reviewed and adjusted by two experienced radiologists to ensure the accuracy of the segmentation. Subsequently, the EAT was automatically segmented by applying an intensity threshold of -190 HU to -30 HU on the epicardium mask. Based on the EAT mask, two quantitative indicators, namely volume and density (CT value) in Hounsfield units (HU), were extracted. Finally, to visually compare EAT between HTN patients and non-HTN participants, three-dimensional reconstructions were generated and displayed from six different perspectives: anterior, posterior, left, right, superior, and inferior.

Moreover, to accurately quantify coronary plaques, CCTA images were utilized to generate plaque masks directly on the three major coronary arteries through deep learning models integrated into the uRP. Specifically, coronary plaques were detected in CCTA images using a detection model, and then were classified into calcified and non-calcified plaques through a classification model. For the regions identified as plaques, they were assigned to the three major coronary arteries, namely the left anterior descending, left circumflex, and right coronary artery. Subsequently, two experienced radiologists reviewed and refined the automatically generated plaque masks on the three major coronary arteries to ensure the accuracy of segmentation and classification. After that, two indicators were extracted from the plaque masks on the three major coronary arteries for evaluation, including plaque volume and coronary artery calcification score (CACS). The CACS was computed as the sum of the product of CT value, area, and weighting coefficient of calcifications with CT values greater than 130 HU across all image layers: CACS = Σ (CT value × area × weighting coefficient).

The computerized tomography-fractional flow reserve (CT-FFR) was also precisely quantified by the uRP. CT-FFR is a non-invasive image post-processing technology. Based on the three-dimensional images of coronary arteries obtained by conventional CCTA, supplemented by the computer-specific software, the calculations can be performed. The details are as follows: according to the coronary artery anatomical model, combined with the mathematical model of coronary physiology and the physical laws of fluid dynamics, the coronary hemodynamics was simulated, the coronary artery blood flow and pressure were obtained, and the CT-FFR at any position of the coronary artery tree was calculated. CT-FFR can be measured at any position of coronary arteries, and the results were presented as color-coded images.

### Measurement of cardiac structure and function

The indicators of cardiac structure and function were examined by echocardiography. According to the recommendations of the American Society of Echocardiography [24], transthoracic echocardiography was performed by two independent, trained and registered cardiac sonographers using Philips iE33 echocardiography system (Koninklijke Philips N.V., Amsterdam, Netherlands). The aorta, right atrium, left atrium, right ventricle, left ventricular end-diastolic diameter, interventricular septum, left ventricular posterior wall and pulmonary artery were measured in the parasternal long-axis view of left ventricle and the parasternal large artery short-axis view, respectively. Besides, left ventricle systolic function was evaluated by calculating left ventricular ejection fraction (LVEF) using the modified Simpson biplanar method. What’s more, LV inflow parameter was obtained by the pulse wave Doppler in the apical four-chamber view, which was the ratio of early diastolic velocity peak to late diastolic velocity peak (E/A ratio).

### Assessment of cardiorenal complications

In this study, both continuous and categorical variables were used to evaluate the situation of cardiorenal complications. In terms of cardiac complications, plaque volume, CACS and CT-FFR were used to evaluate coronary artery condition. At the same time, the indicators of echocardiography and the biochemical indicator NT-proBNP were used to evaluate cardiac structure and function. In terms of renal complications, serum creatinine, BUN, CysC, eGFR, and uACR were used to assess renal function. Furthermore, in terms of categorical variables, cardiac complication was defined as a composite indicator of the presence of coronary artery disease, heart failure and atrial fibrillation, while renal complication was defined as a composite indicator of chronic kidney disease (CKD) stages 3 ~ 5 and uACR > 300 mg/g.

### Statistical analysis

All statistical analyses were performed using SPSS 26.0 (International Business Machines Corp., New York, USA) and R 4.3.0 (R Foundation for Statistical Computing, Vienna, Austria). Firstly, the Kolmogorov-Smirnov test was used to determine whether the data conformed to Gaussian distribution. For continuous variables, if the Gaussian distribution was consistent, they were expressed as mean ± standard deviation (SD), and the t test was used for analysis; otherwise, they were shown as median and interquartile range (IQR), and the Wilcoxon rank-sum test was used. Categorical variables were presented as frequency and percentages, and χ^2^ test was used for analysis. In correlation analysis, the Pearson product-moment correlation coefficient or Spearman’s rank correlation coefficient was used to examine the relationship between EAT characteristics and indicators of cardiorenal complications, applying the Bonferroni correction to adjust for multiple comparisons.

Moreover, mediation analysis was performed according to the AGReMA Statement [25]. HTN, cardiorenal complications indicators, and EAT were included in mediation analysis, with HTN as the independent variable, cardiorenal complication indicators as the dependent variables, and EAT was considered as the mediating variable. At the same time, sex, age, BMI, smoking, drinking, diabetes mellitus, hyperlipidemia, and medications (including antiplatelets, statin, angiotensin converting enzyme inhibitor/angiotensin receptor blocker, angiotensin receptor-neprilysin inhibitor, β-blocker, and sodium-dependent glucose transporter 2 inhibitor) were managed as covariates in analysis. Parametric regression was used to estimate total, indirect, and direct effects between HTN, cardiorenal complications, and EAT. The direct effect refers to the effect of HTN (exposure) on cardiorenal complications (outcome) after adjustment for EAT (mediator), whereas the indirect effect, also known as the mediating effect, represents the product of the effect of HTN on EAT and the effect of EAT on cardiorenal complications. The significant differences in these models would imply that EAT specifically contributes to complementary variability in cardiorenal complications beyond the effect of HTN alone. To further quantify the magnitude of mediating effect, the study estimated the proportion of EAT-mediated associations [(indirect effect) / (direct effect + indirect effect)] if the indirect effect was significant. Confidence intervals (CIs) were calculated using bootstrap resampling and used to assess the significance of mediating effect. All presented βs were standardized regression coefficients and were therefore directly comparable. A two-tailed *P* < 0.05 was considered statistically significant.

## Results

### Characteristics of included patients

**Fig. 2:**
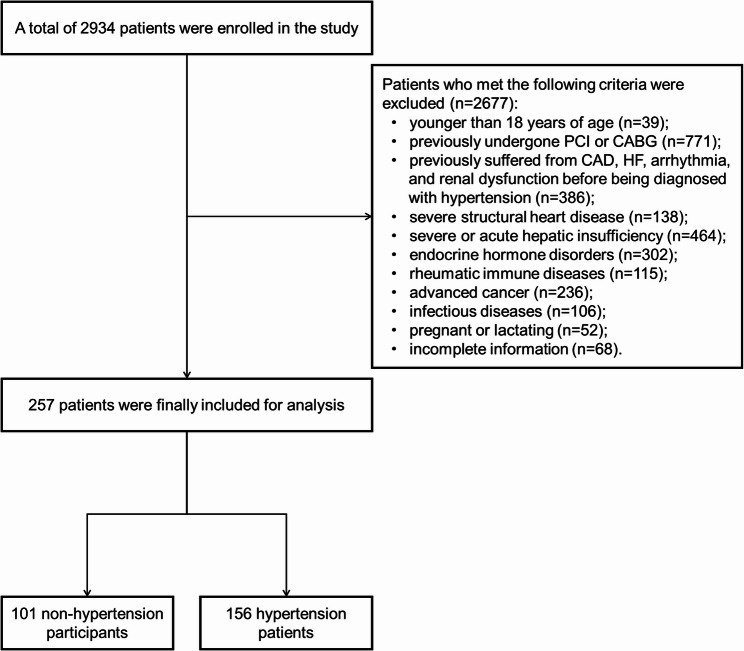
Flow diagram of the study. PCI, percutaneous coronary intervention; CABG, coronary artery bypass grafting; CAD, coronary artery disease; HF, heart failure

The study initially screened 2934 patients from May 2021 through May 2023. As showed in Fig. 2, after excluding 2677 patients based on criteria, a total of 257 patients (156 HTN and 101 non-HTN) were enrolled for analysis. The baseline demographic, clinical characteristics, medications history, and laboratory tests of included patients are summarized in Table 1. Compared to non-HTN participants, HTN patients had higher levels of baseline body mass index (*P* = 0.024), systolic blood pressure (*P* < 0.001), and diastolic blood pressure (*P* < 0.001). However, there was no significant differences in age, sex and other clinical characteristics between two groups (all *P* > 0.05). In terms of medications history, the use of antiplatelets (55.1% vs. 31.7%, *P* < 0.001), statin (70.5% vs. 46.5%, *P* < 0.001), angiotensin converting enzyme inhibitor/angiotensin receptor blocker (51.3% vs. 2.0%, *P* < 0.001), angiotensin receptor-neprilysin inhibitor (9.0% vs. 2.0%, *P* = 0.023), calcium channel blocker (62.2% vs. 2.0%, *P* < 0.001), β-blocker (32.7% vs. 9.9%, *P* < 0.001) and sodium-dependent glucose transporter 2 inhibitor (19.9% vs. 8.9%, *P* = 0.018) in HTN patients was significantly higher than those in non-HTN participants.

**Table 1:** Baseline characteristics of HTN versus non-HTN patients

Variables	Total (*n* = 257)	Non-HTN (*n* = 101)	HTN (*n* = 156)	*P*
Age, year, mean ± SD	63.1 ± 9.5	62.6 ± 10.7	63.5 ± 8.6	0.489
Male, n (%)	170 (66.1)	69 (68.3)	101 (67.4)	0.554
BMI, kg/m^2^, mean ± SD	24.4 ± 3.1	23.8 ± 3.2	24.7 ± 2.9	0.024*
DM, n (%)	85 (33.1)	32 (31.7)	53 (34.0)	0.703
Hyperlipidemia, n (%)	48 (18.7)	13 (12.9)	35 (22.4)	0.055
Smoke, n (%)	80 (31.1)	33 (32.7)	47 (30.1)	0.667
Alcohol, n (%)	50 (19.5)	16 (15.8)	34 (21.8)	0.239
SBP, mmHg, mean ± SD	132.4 ± 18.3	126.5 ± 13.4	136.2 ± 20.1	< 0.001***
DBP, mmHg, mean ± SD	80.5 ± 11.1	77.6 ± 9.6	82.5 ± 11.5	< 0.001***
HR, bpm, median (IQR)	78 (71, 86)	79 (72, 87)	77 (70, 86)	0.114
Hb, g/L, mean ± SD	132.1 ± 19.4	129.7 ± 18.6	133.7 ± 19.7	0.110
FPB, mmol/L, mean ± SD	6.09 ± 2.31	5.81 ± 2.14	6.27 ± 2.40	0.146
HbA1c, %, mean ± SD	6.72 ± 1.61	6.54 ± 1.77	6.82 ± 1.53	0.275
TC, mmol/L, mean ± SD	4.71 ± 1.35	4.73 ± 1.27	4.69 ± 1.41	0.792
TG, mmol/L, mean ± SD	1.64 ± 1.02	1.50 ± 0.83	1.74 ± 1.13	0.072
LDL-C, mmol/L, mean ± SD	2.79 ± 1.11	2.87 ± 1.08	2.74 ± 1.14	0.358
HDL-C, mmol/L, mean ± SD	1.03 ± 0.33	1.00 ± 0.33	1.05 ± 0.33	0.259
Lp(a), mg/L, median (IQR)	159.0 (85.5, 275.0)	161.5 (93.5, 259.0)	156.0 (76.0, 293.0)	0.815
Uric acid, µmol/L, mean ± SD	378.8 ± 113.1	374.6 ± 117.6	381.6 ± 110.2	0.629
Antiplatelets, n (%)	118 (45.9)	32 (31.7)	86 (55.1)	< 0.001***
Statin, n (%)	157 (61.1)	47 (46.5)	110 (70.5)	< 0.001***
ACEI/ARB, n (%)	82 (31.9)	2 (2.0)	80 (51.3)	< 0.001***
ARNI, n (%)	16 (6.2)	2 (2.0)	14 (9.0)	0.023*
CCB, n (%)	99 (38.5)	2 (2.0)	97 (62.2)	< 0.001***
B-blocker, n (%)	61 (23.7)	10 (9.9)	52 (32.7)	< 0.001***
Diuretic, n (%)	26 (10.1)	6 (5.9)	20 (12.8)	0.074
Insulin, n (%)	43 (16.7)	13 (12.9)	30 (19.2)	0.182
Metformin, n (%)	56 (21.8)	16 (15.8)	40 (25.6)	0.063
Sulfonylureas, n (%)	13 (5.1)	5 (5.0)	8 (5.1)	0.949
A-glucosidase inhibitors, n (%)	17 (6.6)	7 (6.9)	10 (6.4)	0.870
DPP-4 inhibitors, n (%)	32 (12.5)	10 (9.9)	22 (14.1)	0.319
GLP-1R agonists, n (%)	5 (1.9)	2 (2.0)	3 (1.9)	0.974
SGLT2i, n (%)	40 (15.6)	9 (8.9)	31 (19.9)	0.018*

*Non-HTN* Non-hypertension, *HTN* Hypertension, *BMI* Body mass index, *DM* Diabetes mellitus, *SBP* Systolic blood pressure, *DBP* Diastolic blood pressure, *HR* Heart rate, *Hb* Hemoglobin, *FPB* Fasting blood glucose, *HbA1c* Glycosylated hemoglobin A1c, *TC* Total cholesterol, *TG* Triglyceride, *LDL-C* Low-density lipoprotein cholesterol, *HDL-C* High-density lipoprotein cholesterol, *Lp(a) *Lipoprotein a, *ACEI* Angiotensin converting enzyme inhibitor, *ARB* Angiotensin receptor blocker, *ARNI* Angiotensin receptor-neprilysin inhibitor, *CCB* Calcium channel blocker, *DPP-4 inhibitors *Dipeptidyl peptidase-4 inhibitors, *GLP-1R agonists* Glucagon-like peptide-1 receptor agonists, *SGLT2i* Sodium-dependent glucose transporter 2 inhibitor, *SD* Standard deviation, *IQR* Interquartile range

* P < 0.05, ** P < 0.01, and *** P < 0.001

### Differences in cardiorenal complications between HTN and non-HTN patients

**Fig. 3:**
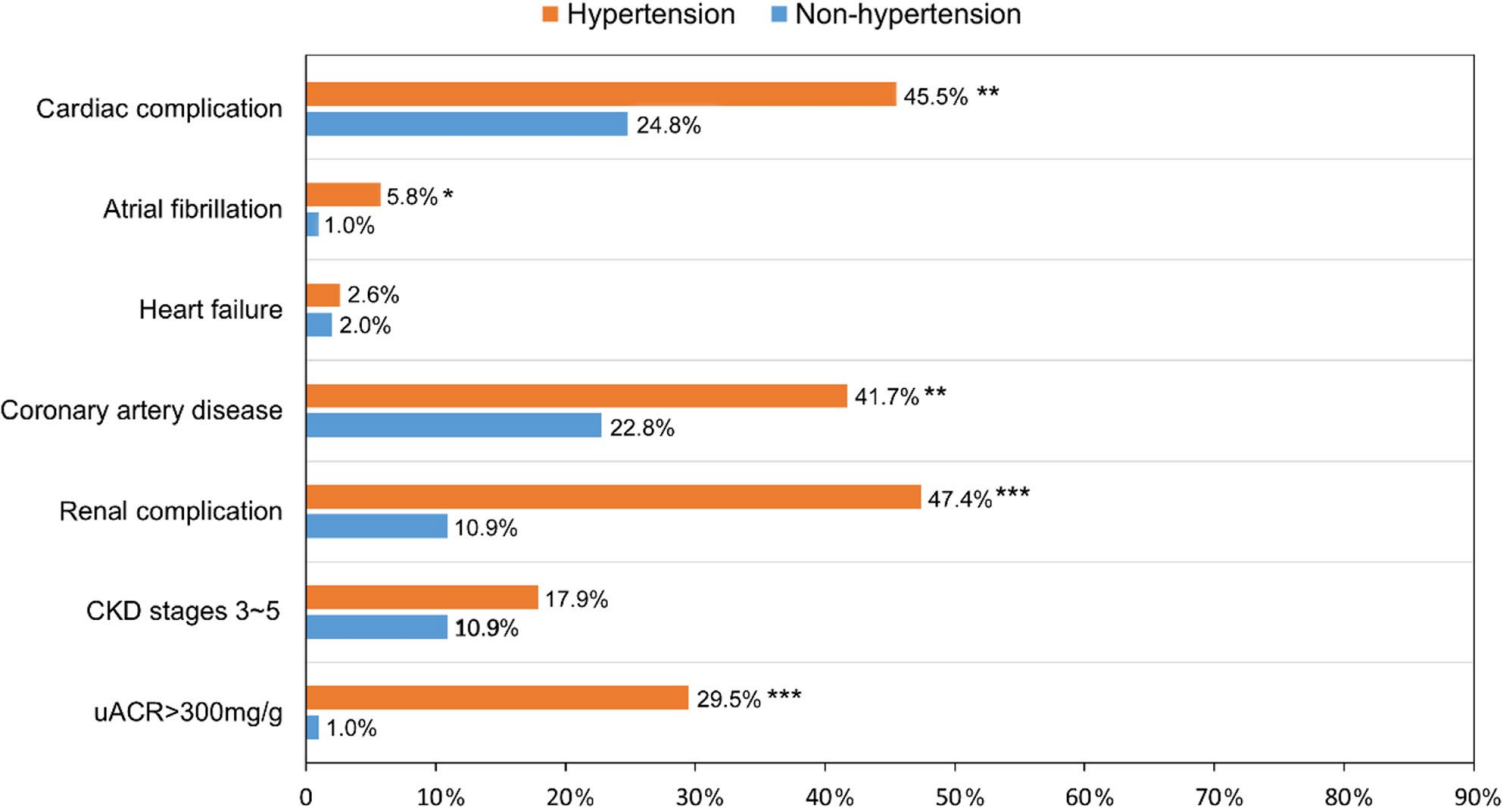
Prevalence of cardiorenal complications between non-HTN and HTN patients. Non-HTN, non-hypertension; HTN, hypertension; CKD, chronic kidney disease; uACR, urinary albumin-to-creatinine ratio. * *P* < 0.05, ** *P* < 0.01, and *** *P* < 0.001

As showed in Table 2, when compared with non-HTN participants, HTN patients had higher levels of CACS [14.9 (0.0, 157.6) vs. 0.0 (0.0, 49.5), *P* < 0.001] and coronary plaque volume [0.0 (0.0, 120.3) vs. 0.0 (0.0, 33.4) mm^3^, *P* = 0.003], with a lower level of CT-FFR (0.88 ± 0.09 vs. 0.92 ± 0.06, *P* < 0.001), indicating that coronary plaque burden was heavier and had a greater impact on blood flow in HTN patients. In addition, HTN patients exhibit more characteristics of myocardial hypertrophy and decreased cardiac function, as manifested by increasing in aorta (*P* = 0.002), left atrium (*P* = 0.008), right atrium (*P* = 0.031), left ventricular end-diastolic diameter (*P* = 0.020), interventricular septum (*P* < 0.001), left ventricular posterior wall (*P* < 0.001), pulmonary artery (*P* < 0.001), and NT-proBNP (*P* = 0.028), with reductions in LVEF (*P* < 0.001) and E/A ratio (*P* = 0.037). Similarly, HTN patients had worse renal function, as evidenced by higher levels of BUN (*P* < 0.001), serum creatinine (*P* < 0.001), CysC (*P* = 0.003), and uACR (*P* < 0.001), and lower level of eGFR (*P* < 0.001). Figure 3 further analyzed the prevalence of complications, it was found that HTN patients typically had a higher prevalence of any cardiac (45.5% vs. 24.8%, *P* = 0.001) and renal (47.4% vs. 10.9%, *P* < 0.001) complications than non-HTN participants, with higher prevalence of coronary artery disease (41.7% vs. 22.8%, *P* = 0.002), atrial fibrillation (5.8% vs. 1.0%, *P* = 0.034), and uACR > 300 mg/g (29.5% vs. 1.0%, *P* < 0.001), respectively.

**Table 2:** Cardiorenal indexes of HTN versus non-HTN patients

Variables	Total (*n* = 257)	Non-HTN (*n* = 101)	HTN (*n* = 156)	*P*
Coronary plaque				
CACS, median (IQR)	2.8 (0.0, 105.2)	0.0 (0.0, 49.5)	14.9 (0.0, 157.6)	< 0.001***
Coronary plaque volume, mm^3^, median (IQR)	0.0 (0.0, 101.3)	0.0 (0.0, 33.4)	0.0 (0.0, 120.3)	0.003**
CT-FFR, mean ± SD	0.90 ± 0.08	0.92 ± 0.06	0.88 ± 0.09	< 0.001***
Cardiac structure and function				
Aorta, mm, mean ± SD	27.9 ± 3.1	27.1 ± 3.1	28.4 ± 2.9	0.002**
RA, mm, mean ± SD	42.4 ± 5.2	42.0 ± 4.8	42.7 ± 5.5	0.370
LA, mm, mean ± SD	32.5 ± 4.6	31.4 ± 4.4	33.1 ± 4.5	0.008**
RV, mm, mean ± SD	22.2 ± 3.2	21.6 ± 3.0	22.6 ± 3.3	0.031*
LVDd, mm, mean ± SD	45.2 ± 5.2	44.2 ± 4.9	45.8 ± 5.3	0.020*
IVS, mm, mean ± SD	10.6 ± 1.6	10.0 ± 1.5	11.0 ± 1.6	< 0.001***
LVPW, mm, mean ± SD	9.5 ± 1.2	9.0 ± 1.0	9.8 ± 1.2	< 0.001***
PA, mm, mean ± SD	22.2 ± 2.3	21.5 ± 2.2	22.6 ± 2.3	< 0.001***
LVEF, %, mean ± SD	63.8 ± 8.7	67.2 ± 6.8	61.8 ± 9.1	< 0.001***
E/A ratio, mean ± SD	0.88 ± 0.38	0.95 ± 0.43	0.84 ± 0.35	0.037*
NT-proBNP, pg/mL, median (IQR)	168.5 (70.0, 307.0)	101.5 (70.0, 306.6)	179.1 (88.5, 305.7)	0.028*
Renal function				
BUN, mmol/L, mean ± SD	6.51 ± 2.64	5.60 ± 1.56	7.09 ± 3.01	< 0.001***
Creatinine, µmol/L, mean ± SD	90.0 ± 66.3	71.6 ± 20.6	101.9 ± 81.4	< 0.001***
CysC, mg/L, mean ± SD	1.23 ± 0.55	1.10 ± 0.27	1.31 ± 0.66	0.003**
eGFR, mL/min/1.73 m^2^, mean ± SD	83.9 ± 26.9	92.1 ± 28.2	78.6 ± 24.7	< 0.001***
uACR, mg/g, mean ± SD	142.7 ± 123.6	34.2 ± 31.5	213.0 ± 109.3	< 0.001***

*Non-HTN* Non-hypertension, *HTN* Hypertension, *CACS* Coronary artery calcification score, *CT-FF*R Computerized tomography-fractional flow reserve, *RA* Right atrium, *LA* Left atrium, *RV* Right ventricle, *LVD*d Left ventricular end-diastolic diameter, *IVS* Interventricular septum, *LVPW* Left ventricular posterior wall, *PA* Pulmonary artery, *LVEF* Left ventricular ejection fraction, *NT-proBNP* N-terminal pro-B-type natriuretic peptide, *BUN* Blood urea nitrogen, *CysC* Cystatin C, *eGFR *Estimated glomerular filtration rate, *uACR* Urinary albumin-to-creatinine ratio, *SD* Standard deviation, *IQR* Interquartile range

* *P* < 0.05, ** *P* < 0.01, and *** *P* < 0.001

### Differences in EAT characteristics between HTN and non-HTN patients

**Fig. 4:**
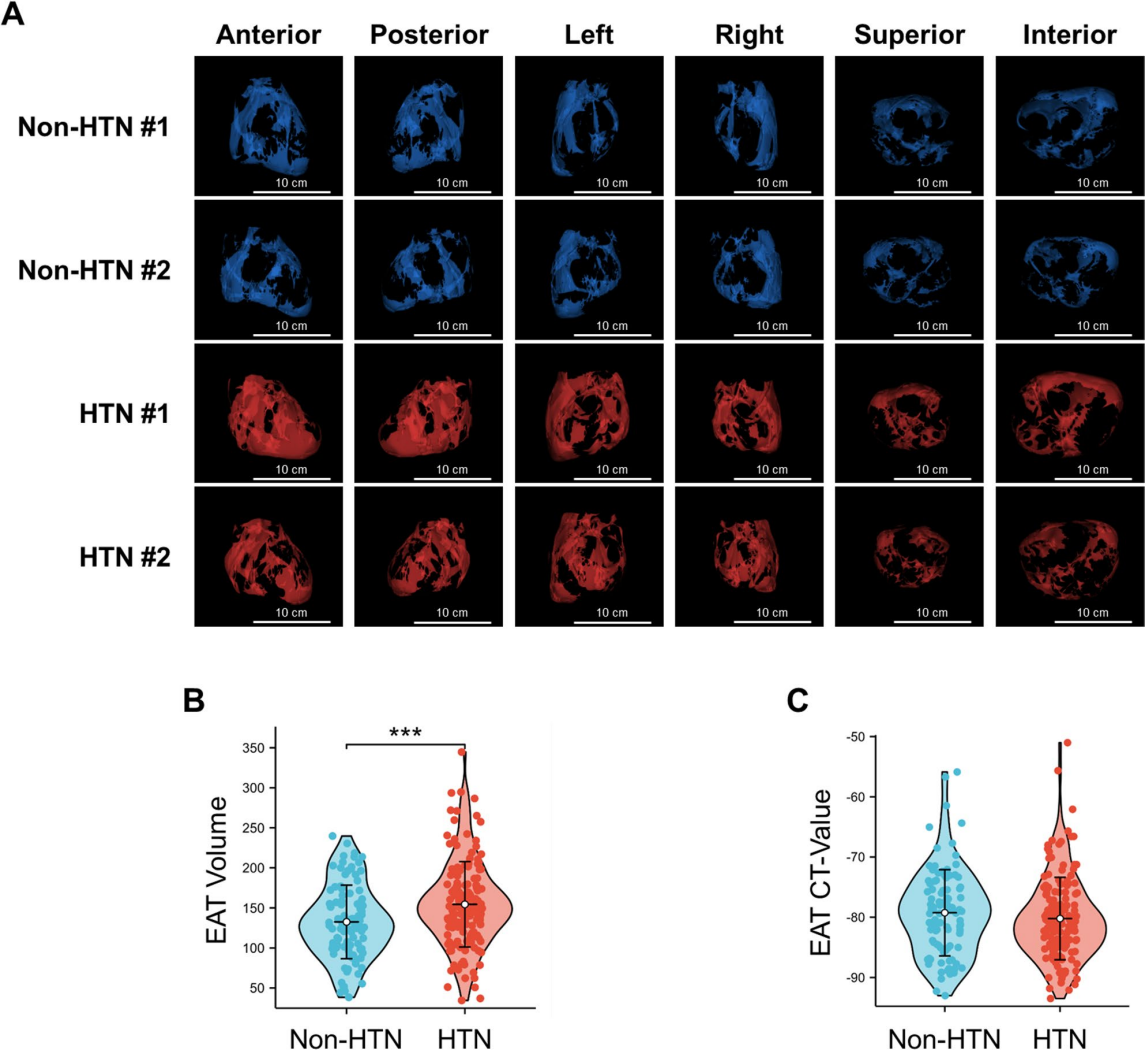
Comparison of EAT characteristics between non-HTN and HTN patients. The 3D reconstructions of EAT from six angles (**A**), as well as statistical plots comparing EAT volume (**B**) and density (**C**) between non-HTN and HTN patients. Scale bar = 10 cm; EAT, epicardial adipose tissue; Non-HTN, non-hypertension; HTN, hypertension; * *P* < 0.05, ** *P* < 0.01, and *** *P* < 0.001

The 3D reconstructions of EAT in HTN and non-HTN patients were displayed from six perspectives in Supplementary Videos and Fig. 4A: anterior, posterior, left, right, superior and interior. Compared with that of non-HTN participants, EAT volume was significantly higher in HTN patients (154.40 ± 53.31 vs. 132.49 ± 45.88 cm^3^, *P* < 0.001, Fig. 4B). Nevertheless, there was no significant difference in CT value between two groups (-80.22 ± 6.84 vs. -79.25 ± 7.16 HU, *P* = 0.279, Fig. 4C), indicating that the density of EAT did not differ markedly in HTN patients.

### Correlations between EAT characteristics and cardiorenal complications in HTN patients

**Fig. 5:**
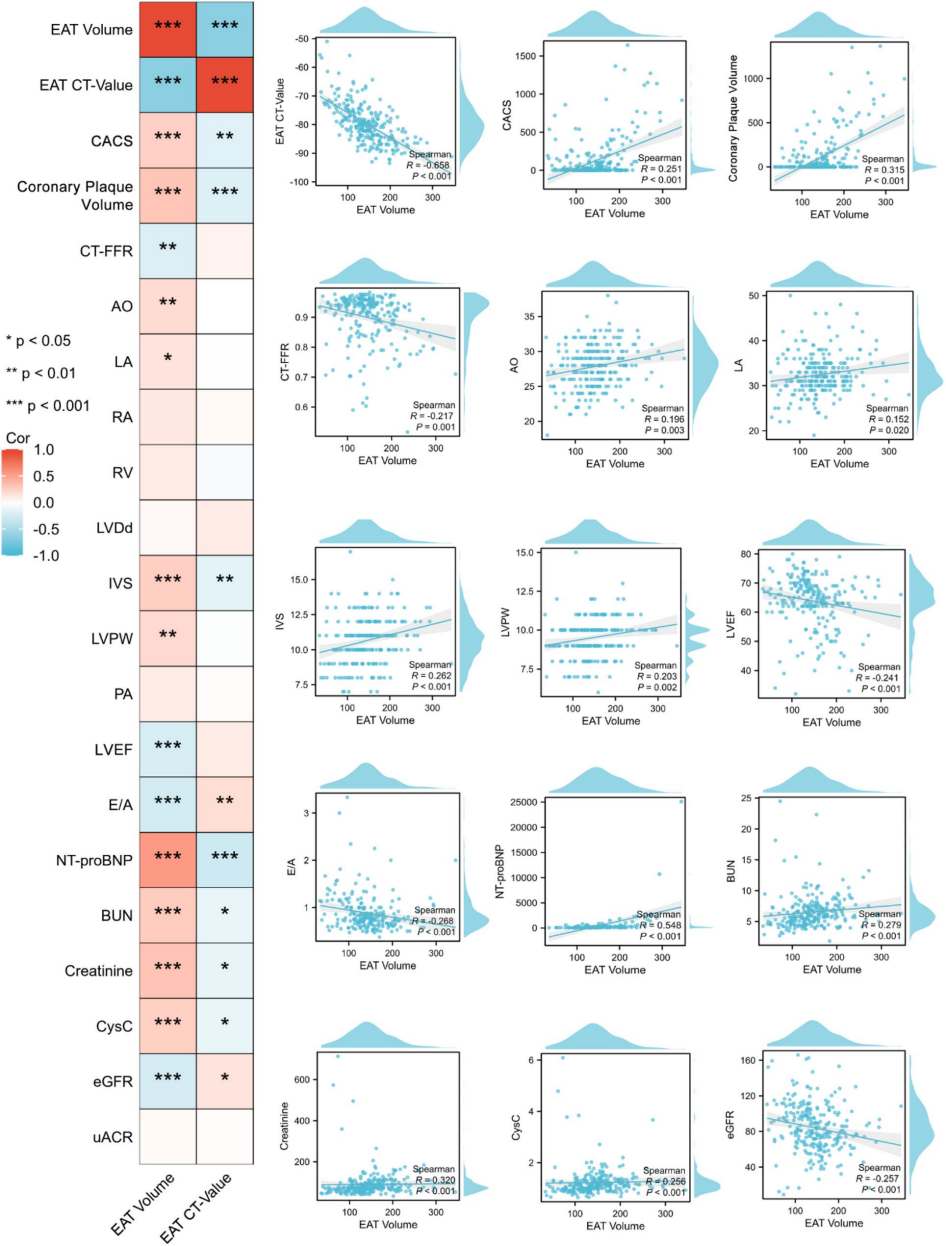
Correlation analysis between EAT characteristics and cardiorenal indicators in HTN patients. Statistical plots of the correlation analysis between the volume and density of EAT and various cardiac and renal indicators in HTN patients. EAT, epicardial adipose tissue; HTN, hypertension; CACS, coronary artery calcification score; CT-FFR, computerized tomography-fractional flow reserve; AO, aorta; LA, left atrium; RA, right atrium; RV, right ventricle; LVDd, left ventricular end-diastolic diameter; IVS, interventricular septum; LVPW, left ventricular posterior wall; PA, pulmonary artery; LVEF, left ventricular ejection fraction; NT-proBNP, N-terminal pro-B-type natriuretic peptide; BUN, blood urea nitrogen; CysC, cystatin C; eGFR, estimated glomerular filtration rate; uACR, urinary albumin-to-creatinine ratio. * *P* < 0.05, ** *P* < 0.01, and *** *P* < 0.001

As shown in Fig. 5, correlation analysis showed that EAT volume was strongly correlated with most indicators of cardiorenal complications (all *P* < 0.05). Among them, EAT volume was found to be significantly associated with the severity of coronary artery disease (increasing CACS and coronary plaque volume, with decreasing CT-FFR, all *P* < 0.05), cardiac hypertrophy and dysfunction (increasing aorta, left atrium, interventricular septum, left ventricular posterior wall, and NT-proBNP, with decreasing LVEF and E/A ratio, all *P* < 0.05), and renal dysfunction (increasing BUN, serum creatinine, and CysC, with decreasing eGFR, all *P* < 0.05). In addition, the CT value of EAT was correlated with partial cardiorenal complications indicators, including CACS, coronary plaque volume, interventricular septum, E/A ratio, NT-proBNP, BUN, serum creatinine, CysC, and eGFR (all *P* < 0.05).

**Fig. 6:**
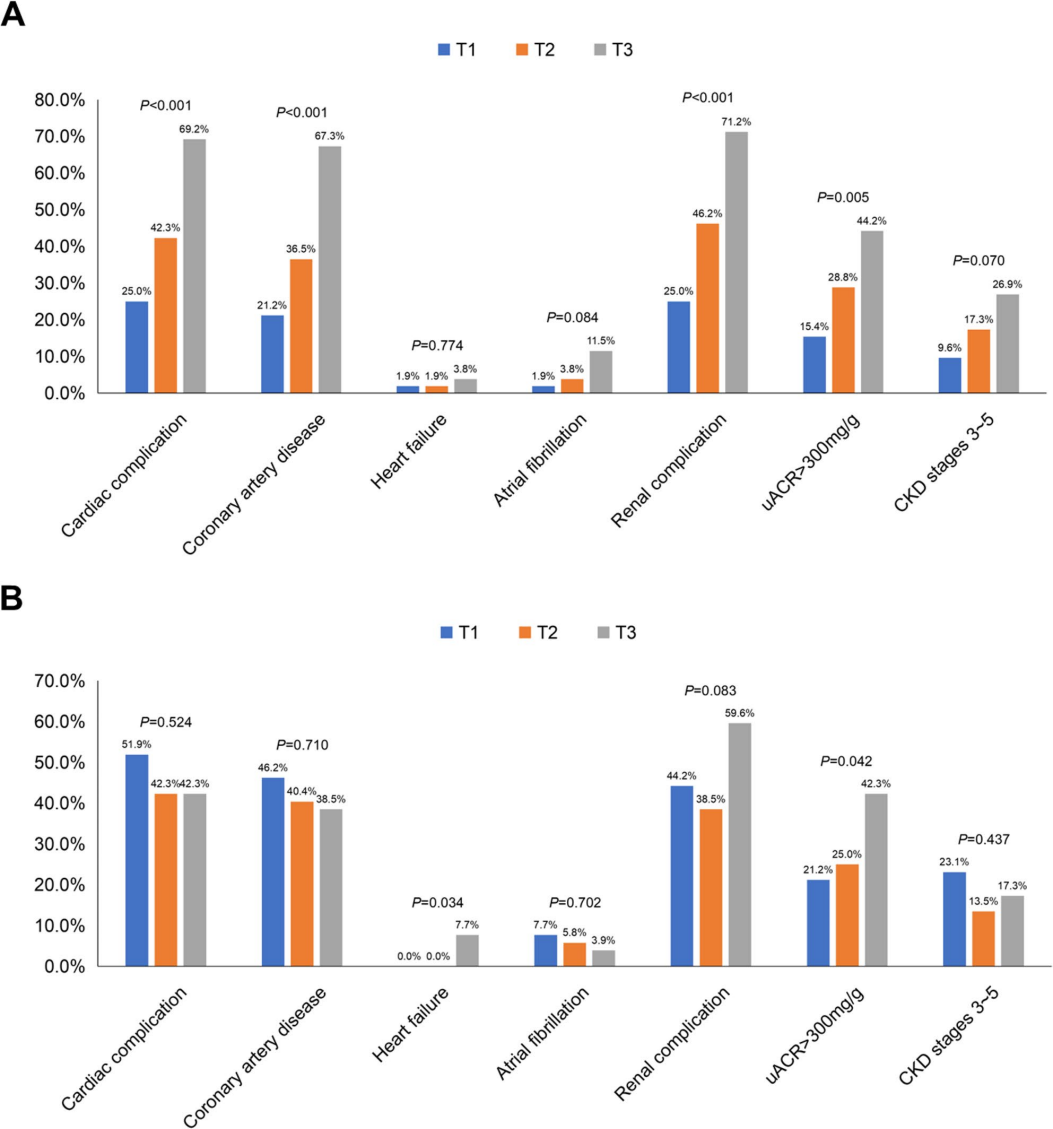
Prevalence of cardiorenal complications in HTN patients according to EAT characteristics tertiles. Bar chart of the prevalence of cardiac and renal complications among HTN patients divided into tertiles based on EAT volume (**A**) and density (**B**). HTN, hypertension; EAT, epicardial adipose tissue; uACR, urinary albumin-to-creatinine ratio; CKD, chronic kidney disease

Subsequently, HTN patients were stratified into tertiles according to EAT volume and density for further analysis. As shown in Fig. 6A, increasing EAT volume tertiles were associated with a significantly higher prevalence of both cardiac (25.0% vs. 42.3% vs. 69.2%, *P* < 0.001) and renal (25.0% vs. 46.2% vs. 71.2%, *P* < 0.001) complications. Specifically, among cardiac complications, this increase was driven by a higher prevalence of coronary artery disease (67.3% vs. 36.5% vs. 21.2%, *P* < 0.001). For renal complications, it corresponded to an increased prevalence of uACR > 300 mg/g (44.2% vs. 28.8% vs. 15.4%, *P* = 0.005). Conversely, Fig. 6B shows that increasing EAT density tertiles were not associated with significant changes in the overall prevalence of cardiac (51.9% vs. 42.3% vs. 42.3%, *P* = 0.524) or renal (44.2% vs. 38.5% vs. 59.6%, *P* = 0.083) complications, although it only manifested as an increase in prevalence of heart failure (0.0% vs. 0.0% vs. 7.7%, *P* = 0.034) and uACR > 300 mg/g (21.2% vs. 25.0% vs. 42.3%, *P* = 0.042).

### Mediation analysis of EAT volume and cardiorenal complication indicators

**Fig. 7:**
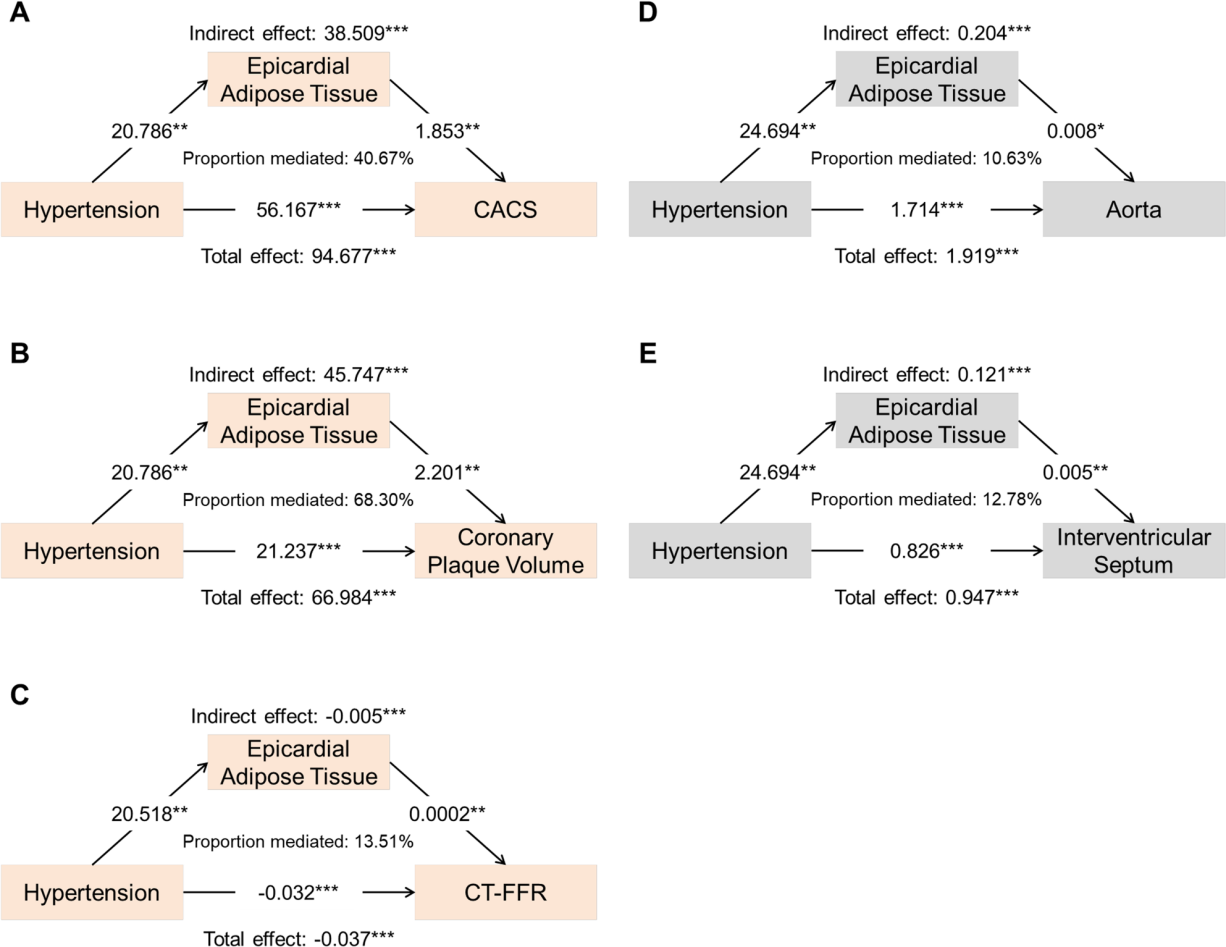
Mediation analysis of the effects of EAT volume on cardiorenal complications. Summary plots of the significant mediating effects of EAT volume between HTN and coronary plaque (**A**, **B** and **C**) and cardiac structure indicators (**D** and **E**), after adjustment for sex, age, BMI, smoke, alcohol, DM, hyperlipemia, and medications (antiplatelets, statin, ACEI/ARB, β-blocker, and SGLT2i). EAT, epicardial adipose tissue; HTN, hypertension; CACS, coronary artery calcification score; CT-FFR, computerized tomography-fractional flow reserve; BMI, body mass index; DM, diabetes mellitus; ACEI, angiotensin converting enzyme inhibitor; ARB, angiotensin receptor blocker; SGLT2i, sodium-dependent glucose transporter 2 inhibitor. * *P* < 0.05, ** *P* < 0.01, and *** *P* < 0.001

Based on the above analysis results, EAT volume and cardiorenal complications indicators were acted as the mediator and dependent variables, respectively, and HTN was added as the independent variable for further mediation analysis. The results of mediation analysis are presented in Supplementary Table 1. It was found that EAT played mediating effects in five mediation models, as summarized in Fig. 7. The results showed that, after multiple mediation analysis based on the adjusted structural equation modeling, EAT volume still had mediating effects between HTN and CACS (β: 38.509; 95% CI: 6.205, 88.020; mediation proportion: 40.67%), plaque volume (β: 45.747; 95% CI: 9.301, 94.330; mediation proportion: 68.30%), CT-FFR (β: -0.005; 95% CI: -0.0150, -0.0001; mediation proportion: 13.51%), aorta (β: 0.204; 95% CI: 0.006, 0.495; mediation proportion: 10.63%), and interventricular septum (β: 0.121; 95% CI: 0.008, 0.300; mediation proportion: 12.78%).

## Discussion

Based on available evidence, this study represents the first investigation into the effects of EAT characteristics on the relationship between HTN and cardiorenal complications. Consistent with observations in other cardiovascular diseases [26], it was found that HTN patients exhibited greater EAT volumes compared to controls, though EAT density did not differ significantly. Moreover, the study found that EAT had mediating effects between HTN and cardiac complications, suggesting that EAT accumulation may contribute to adverse cardiac outcomes in HTN patients.

HTN constitutes a major global health burden. Driven by societal aging and environmental shifts, HTN prevalence continues to rise annually, establishing it as one of the most prevalent chronic conditions in China, Europe, and worldwide [27, 28]. Pathophysiologically, HTN involves complex interactions among the renin-angiotensin-aldosterone system, natriuretic peptides, endothelial function, sympathetic nervous system activity, and immune responses [29]. As the disease progresses, it ultimately induces target-organ damage, leading to diverse complications. Therefore, it is very necessary to pay attention to HTN in order to find better methods for monitoring HTN and cardiorenal complications.

EAT, as a brown adipose tissue (BAT), is generally recognized for its protective, paracrine, and metabolic effects on the myocardium. However, under conditions like obesity or diabetes mellitus, EAT undergoes a phenotypic shift toward a pro-inflammatory state [30]. In this state, EAT secretes inflammatory cytokines, including tumor necrosis factor-α (TNF-α) and interleukin-6 (IL-6), which can promote chronic myocardial inflammation, exert detrimental effects on cardiomyocytes, and contribute to cardiovascular pathogenesis [30, 31].

Advances in imaging technology, particularly CCTA, now enable accurate quantification of both EAT volume and density [32]. Crucially, emerging evidence suggests that EAT volume and density may exert distinct pathophysiological influences on cardiorenal complications. Increased EAT volume primarily associates with systemic inflammation and adverse biomechanical effects on cardiac and renal function [33, 34]. Conversely, reduced EAT density, which reflects elevated lipid content and impaired thermogenic activity, demonstrates a more specific association with metabolic dysregulation and the development of cardiorenal complications [34].

Nevertheless, as one of the common cardiovascular diseases, the relationship between HTN and EAT remains debated, and the precise associations between EAT characteristics and cardiorenal complications are still being defined. More critically, it remains unresolved which of the two clinically significant EAT characteristics, volume or density, holds greater prognostic relevance in HTN patients. Thus, characterizing both EAT volume and density via CCTA may offer a potential method for monitoring the occurrence and development of HTN and, significantly, for stratifying risk and understanding the mechanisms underlying cardiorenal complications.

To solve this problem, this study focused on EAT, HTN, and cardiorenal complications. The CCTA, echocardiography, and laboratory tests were used to evaluate the characteristics of EAT and various cardiorenal complication indicators in multiple dimensions. This study found that EAT was higher in HTN patients compared with non-HTN patients. However, these results were inconsistent with the study by Shim IK et al. [35]. There may be the following reasons. First, a key limitation is that their analysis was stratified by sex. While it compared EAT between HTN and non-HTN groups within and across sex categories, it did not evaluate these differences in the entire study population. The key factor of sex may have biased the results somewhat. Next, some studies have shown that estrogen was a non-negligible factor for EAT [36, 37]. Estrogen can affect the metabolism of adipocytes through estrogen receptor and reduce the level of inflammatory factors in adipocytes, thereby affecting EAT. Nonetheless, Shim IK et al.’s study [35] did not exclude pregnant, lactating and postmenopausal female patients, in whom estrogen expression level may become a confounding factor for the association between EAT and HTN.

Currently, some studies have shown that when the body was in obesity, DM, or metabolic syndrome, EAT would be whitening, in other words, it would be transformed into white adipose tissue [38–41]. Unexpectedly, as a kind of BAT, although this study found that EAT was higher in HTN patients, the density of EAT did not change. Despite HTN is often accompanied by metabolic abnormalities and obesity, which may cause whitening in other adipose tissues, EAT has remained relatively stable in its brown adipose properties. There may be several reasons. Firstly, EAT has a high metabolic rate, its adipocytes are rich in mitochondria and have a strong thermogenic capacity [42]. Since the heart is a high-energy-consuming organ, EAT may be required to maintain the high metabolic activity to support the energy of the heart, and this activity may continue even in the setting of HTN [43]. Thus, the brown adipose properties of EAT, such as hypermetabolic and thermogenic functions, are essential for heart health and may enable it to preserve as BAT without readily whitening. Secondly, in HTN, EAT may play a role in regulating blood pressure to a certain extent, including by secreting fatty acids and cytokines to influence local vascular function [44, 45]. Studies showed that EAT was related to the cardiac hemodynamics, and can help reduce the pressure on blood vessels by secreting adipokines [46, 47]. In this case, EAT retains the brown adipose properties and may be helpful to regulate cardiac function, thereby preventing or mitigating the adverse effects of HTN. Thirdly, HTN patients are usually accompanied by a certain degree of chronic low-grade inflammation [48], which may have a certain impact on the whitening process of EAT. However, the inflammatory response in EAT may be more localized, and because of its special anatomical location, may be less obvious or less intense than in adipose tissue elsewhere [49–51]. For this reason, EAT may not undergo whitening as dramatically as abdominal adipose tissue. Furthermore, some technical factors influencing HU measurements, such as CT acquisition parameters and partial volume effects, may also influence EAT density results.

Interestingly, this study found that EAT partially mediated the relationship between HTN and cardiac complications, but did not play a significant mediating effect in renal complications. As a heart-specific visceral depot in direct contact with coronary arteries and myocardium, EAT can shift from a protective, brown-like phenotype to a pro-inflammatory, lipotoxic state under HTN, driven by reduced adiponectin signaling and dysregulated SIRT1-NF-κB activity [52]. This shift increases local secretion of IL-6, TNF-α and other mediators that promote endothelial dysfunction, vascular stiffening and adverse myocardial remodeling [53]. Concurrent renin-angiotensin-aldosterone system (RAAS) activation, oxidative stress and pro-inflammatory macrophage polarization amplify paracrine injury to endothelium and myocardium, accelerating atherosclerosis and plaque vulnerability [54–58]. Furthermore, hypertensive EAT also releases excess free fatty acids and metabolites that induce oxidative damage and metabolic stress within the coronary-myocardial unit, while EAT hyperplasia and remodeling contribute to myocardial hypertrophy, fibrosis and even mechanical coronary compression [59–63]. These convergent metabolic, inflammatory and mechanical effects explain why EAT volume, a marker of total pro-inflammatory/lipotoxic burden, rather than density, more robustly mediates hypertensive organ damage. Importantly, the adipose-vascular remodeling is modifiable: lipid-lowering and metabolic interventions can improve vascular mechanics, supporting a unified vascular-adipose paradigm for therapeutic targeting [64]. Finally, EAT-driven microenvironmental changes can also disturb myocardial electrophysiology, further linking EAT expansion to clinically relevant cardiac outcomes [65]. On the contrary, although the incidence of renal complications is second only to cardiac complications in HTN complications [4], due to the anatomical location of the kidney being far away from EAT, the direct effect of EAT on the kidney is relatively limited. Besides, the pathological mechanism of the two is different. One primarily relies on the hemodynamic changes of the kidney [66, 67], and the other is mainly dependent on EAT’s intense metabolic and inflammatory activity. Therefore, the mediating effects of EAT are more concentrated on cardiac complications, while there is no significant mediating effect between HTN and renal complications.

Notably, the increased frequency of sodium-dependent glucose transporter 2 (SGLT2) inhibitors use in the HTN cohort presents a potential confounding factor. Recent studies indicate that SGLT2 inhibitors exert beneficial effects on EAT and provide cardiorenal protection through various pathways, including hemodynamic improvement, anti-inflammatory action, and direct renoprotection [68, 69]. Consequently, their widespread use may have attenuated the observed associations between EAT and HTN complications in this study. To mitigate potential bias, the use of SGLT2 inhibitors was included as a covariate in the mediation analysis to enhance the validity of the findings. However, the possibility of residual confounding must be acknowledged as a limitation of the study.

Building on the findings that EAT contributes to HTN-related cardiorenal complications, emerging therapeutic strategies targeting this specific visceral adipose tissue may provide novel avenues to reduce cardiometabolic risk. Beyond lifestyle modification, lipid-lowering and metabolic interventions have shown potential in mitigating EAT accumulation and inflammation. Nowadays, RNA-based lipid-lowering strategies have been shown to impact not only LDL-C but also vascular risk profiles and mechanical properties, exemplified by Inclisiran, an siRNA that inhibits hepatic PCSK9 synthesis and exerts substantial effects on lipid metabolism and systemic inflammation [70]. Similar to Inclisiran, SGLT2 inhibitors also demonstrate pleiotropic benefits in improving endothelial function and reducing vascular stiffness [69]. By modulating these pathways, such therapies may indirectly influence EAT and its downstream cardiovascular effects, thereby positioning EAT as a promising translational target within precision cardiometabolic medicine.

## Strengths and Limitations

This study has several notable strengths. First, it offers a comprehensive and multidimensional assessment of EAT by simultaneously assessing both volume and density using high-resolution CCTA, enabling a direct comparison of their clinical relevance in HTN. Second, the integration of AI-assisted segmentation with verification by expert radiologists ensures robust and reproducible quantification of EAT and coronary plaque burden. Third, this study systematically correlates EAT characteristics with a broad range of cardiac and renal structural, functional, and biochemical markers, providing an integrated perspective on hypertensive target-organ damage. Finally, through mediation analysis with extensive adjustment for clinical confounders, the study strengthens the mechanistic interpretation of EAT volume as a potential intermediary linking HTN to adverse cardiac outcomes, thereby enhancing the translational relevance of findings.

There are still some limitations in this study. Firstly, the study’s single-center, cross-sectional design precludes causal inference and allows for unmeasured confounding or reverse causality, so it cannot determine whether higher EAT volume is a cause, consequence, or merely a marker of more advanced hypertensive disease. Secondly, previous studies have established ethnic differences in body composition, adipose tissue distribution, and metabolic regulation [71], suggesting potential population-specific variations in EAT characteristics. As this study was conducted exclusively in a Chinese cohort and lacked waist circumference, visceral adiposity, or insulin-resistance indices (e.g., HOMA-IR, TyG, TG/HDL), which are all strongly associated with EAT burden and target-organ damage, caution is warranted when extrapolating these results to other ethnic populations. Furthermore, while this study establishes EAT as a mediator between HTN and cardiac complications, its specific molecular mechanisms remain undefined. Future in-depth studies should therefore elucidate these pathways and validate the findings in multi-ethnic cohorts. To this end, the implementation of harmonized EAT quantification protocols is essential for robust comparisons.

## Conclusion

In this study, EAT volume was a superior predictor of cardiorenal complications compared to its density in HTN patients. Furthermore, EAT was identified to mediate the relationship between HTN and cardiac complications, especially with coronary artery disease and heart failure. This finding is of great value in helping to develop effective interventions to prevent the occurrence and development of cardiac complications in HTN patients by targeting heart-specific visceral adipose tissue.

## Supplementary Information

Supplementary Material 1. Supplementary Videos

Supplementary Material 2. Supplementary Methods

Supplementary Material 3. Supplementary Tables

## Data Availability

The datasets generated and/or analyzed during the current study are available from the corresponding author upon reasonable request.
