# Supplementary material for: Epicardial adipose tissue volume outperforms density in association with cardiorenal complications in hypertensive patients
Source: Lipids Health Dis. 2026 Feb 12;25:82. doi: 10.1186/s12944-026-02873-x (PMC12998057; doi:10.1186/s12944-026-02873-x)
Supplement: Supplementary file 2 — Supplementary Material 2. Supplementary Methods [file 12944_2026_2873_MOESM2_ESM.pdf]

Supplementary Methods

1. CCTA examination

All coronary computerized tomography angiography (CCTA) examinations were performed using the Revolution Apex 512-slice CT scanner (GE Healthcare Technologies, Inc., Illinois, USA). Before the examination, patients received 0.5 mg of sublingual nitroglycerin to induce vasodilation and were placed in a supine position, with lead shielding applied to the pelvic region for protection. The CT scan was configured with the following parameters: Cardiac scan mode, manual tube voltage of 100 kV, tube current in smart mA mode (400-600 mA range), noise index of 11, 256×0.625 mm collimation, reconstructed slice thickness of 0.625 mm with a 0.5 mm interval, 280 ms rotation time, and matrix of 512×512 pixels. Threshold triggering was implemented by placing a region of interest (ROI) in the descending aorta, with a trigger threshold set to 65 Hounsfield units (HU). The scan coverage spanned from 1 cm distal to the tracheal bifurcation to the transverse diaphragm. Motion artifacts were corrected using second-generation snapshot freeze technology. Ioversol, as the contrast agent, was infused at a rate of 5 ml/s for a total volume of 60 ml, followed by 30 ml saline administered at the same rate.

2. EAT measurement

The precise delineations of epicardial adipose tissue (EAT) volume boundaries were performed through collaboration between experienced radiologists and intelligent segmentation tools, that was, the uAI Research Portal (United Imaging Intelligence Co., Ltd., Shanghai, China). Meanwhile, annotation tools included ITK-SNAP 3.8.0 (Penn Image Computing and Science Laboratory of University of Pennsylvania, Pennsylvania, USA) and 3D Slicer 5.0.2 (Brigham and Women's Hospital of Harvard Medical School, Massachusetts, USA). The annotation process was initially conducted by two junior radiologists (Liu M and Mao W), followed by rigorous review and optimization by a senior radiologist (Qin J) to ensure the accuracy. Automatic epicardial segmentation was performed using the deep learning model, namely nnUNet. The segmentation model applied HU thresholds ranging from -30 to -190 for pixel values, with all datasets undergoing at least three rounds of head-to-head iterations to ensure precision.

3. Coronary plaque measurement

The measurements of CACS, coronary plaque volume, and CT-FFR were conducted using CCTA image data, with strict adherence to image quality standards to ensure suitability for analysis. The annotation process was systematically carried out in two stages: initial annotations were performed by two junior radiologists (Liu M and Mao W), followed by comprehensive review and refinement by a senior radiologist (Meng Z) to guarantee the quality. The process employed annotation tools, including ITK-SNAP 3.8.0 (Penn Image Computing and Science Laboratory of University of Pennsylvania, Pennsylvania, USA) and 3D Slicer 5.0.2 (Brigham and Women's Hospital of Harvard Medical School, Massachusetts, USA), complemented by intelligent segmentation using the nnUNet.

3.1. CACS calculation

The coronary artery calcification score (CACS) serves as a quantitative measure of coronary artery calcification severity and represents an important indicator for assessing the progression of coronary atherosclerosis. It was calculated based on CCTA images as follows:

(1) Calcification identification: CCTA images were used to identify calcified regions in coronary arteries, with calcification defined as regions demonstrating CT values exceeding 130 HU.

(2) Area and CT value measurement: The area and CT value of each calcified region were measured.

(3) CACS calculation: The CACS was calculated using the formula:

$$\text{CACS} = \Sigma\ (\text{CT value} \times \text{area} \times \text{weighting coefficient})$$

For calcified regions with CT value greater than 130 HU, the sum of the products of CT value, area and weighting coefficient constituted CACS.

3.2. Coronary plaque volume calculation

The coronary plaque volume serves as a crucial indicator for assessing the severity of coronary artery plaques, representing another essential indicator in the evaluation of coronary artery disease. It was quantified using CCTA images through the following steps:

(1) Plaque identification: The identification of plaque regions within the coronary arteries was accomplished using CCTA images, where a deep learning model was used to differentiate between calcified and non-calcified plaques.

(2) Plaque segmentation: Identified plaque regions were segmented to generate corresponding plaque masks.

(3) Volume calculation: The volume of each plaque was calculated using the formula:

$$\text{plaque volume} = \Sigma\ (\text{plaque mask pixel values} \times \text{voxel volume})$$

The voxel volume was determined based on the resolution of the CT scan.

3.3. CT-FFR quantification

The computerized tomography-fractional flow reserve (CT-FFR) represents a non-invasive imaging technology that enables post-processing of CCTA image data. It enables the measurement of coronary arteries at any position, with results displayed as color-coded images, facilitating clinical assessment of the impact of coronary stenosis on blood flow. Based on the three-dimensional coronary artery images obtained from CCTA, the following steps were performed:

(1) Anatomical model construction: A highly detailed three-dimensional reconstruction of the coronary arteries was generated, based on its anatomical structure and spatial relationships.

(2) Hemodynamic simulation: By integrating a mathematical model of coronary physiology with laws of fluid dynamics, coronary hemodynamics was simulated.

(3) Pressure and flow calculation: The blood flow pattern and pressure distribution within coronary arteries, were derived from the hemodynamic simulation.

(4) CT-FFR calculation: The CT-FFR was calculated throughout the entire coronary artery tree, with results presented as color-coded images.
