# Supplementary material for: Epicardial adipose tissue volume outperforms density in association with cardiorenal complications in hypertensive patients
Source: Lipids Health Dis. 2026 Feb 12;25:82. doi: 10.1186/s12944-026-02873-x (PMC12998057; doi:10.1186/s12944-026-02873-x)
Supplement: Supplementary file 3 — Supplementary Material 3. Supplementary Tables [file 12944_2026_2873_MOESM3_ESM.pdf]

Supplementary Table 1 Mediation analysis between HTN and cardiorenal complications via EAT volume.

Dependent variable	β (Bootstrap 95% CI)		Mediation proportion
	Crude	Adjusted[a]	
**Coronary plaque**			
**CACS**			
Total effect	101.007 (34.742, 167.271)	94.677 (15.438, 173.915)	
Direct effect	54.471 (-7.520, 116.462)	56.167 (-18.966, 131.301)	
Indirect effect	46.535 (15.453, 88.819)	38.509 (6.205, 88.020)	40.67%
**Coronary plaque volume**			
Total effect	73.651 (15.269, 132.033)	66.984 (-3.613, 137.580)	
Direct effect	21.982 (-29.339, 73.303)	21.237 (-41.692, 84.165)	
Indirect effect	51.669 (19.468, 92.261)	45.747 (9.301, 94.330)	68.30%
**CT-FFR**			
Total effect	-0.037 (-0.059, -0.015)	-0.037 (-0.062, -0.013)	
Direct effect	-0.030 (-0.052, -0.008)	-0.032 (-0.056, -0.007)	
Indirect effect	-0.007 (-0.0151, -0.001)	-0.005 (-0.0150, -0.0001)	13.51%
**Cardiac structure and function**			
**Aorta**			
Total effect	1.297 (0.495, 2.098)	1.919 (0.939, 2.898)	
Direct effect	1.041 (0.226, 1.857)	1.714 (0.724, 2.704)	
Indirect effect	0.255 (0.051, 0.516)	0.204 (0.006, 0.495)	10.63%
**RA**			
Total effect	0.645 (-0.769, 2.059)	0.807 (-0.898, 2.511)	
Direct effect	0.199 (-1.244, 1.643)	0.439 (-1.287, 2.165)	
Indirect effect	0.446 (0.023, 1.018)	0.368 (-0.028, 0.948)	-
**LA**			
Total effect	1.639 (0.438, 2.840)	2.280 (0.823, 3.737)	
Direct effect	1.370 (0.139, 2.601)	2.043 (0.564, 3.521)	
Indirect effect	0.269 (-0.055, 0.704)	0.237 (-0.079, 0.703)	-

**RV**			
Total effect	0.940 (0.088, 1.793)	1.172 (0.096, 2.247)	
Direct effect	0.825 (-0.053, 1.703)	1.027 (-0.066, 2.120)	
Indirect effect	0.115 (-0.118, 0.373)	0.145 (-0.075, 0.450)	-
**LVDd**			
Total effect	1.628 (0.253, 3.002)	3.069 (1.439, 4.699)	
Direct effect	1.658 (0.240, 3.077)	3.092 (1.428, 4.757)	
Indirect effect	-0.031 (-0.433, 0.401)	-0.024 (-0.426, 0.402)	-
**IVS**			
Total effect	0.979 (0.558, 1.401)	0.947 (0.425, 1.468)	
Direct effect	0.831 (0.404, 3.077)	0.826 (0.300, 1.351)	
Indirect effect	0.148 (0.035, 0.298)	0.121 (0.008, 0.300)	12.78%
**LVPW**			
Total effect	0.777 (0.466, 1.088)	0.753 (0.369, 1.137)	
Direct effect	0.705 (0.386, 1.023)	0.687 (0.297, 1.077)	
Indirect effect	0.072 (0.002, 0.163)	0.066 (-0.004, 0.166)	-
**PA**			
Total effect	1.030 (0.428, 1.631)	1.444 (0.689, 2.199)	
Direct effect	1.005 (0.384, 1.625)	1.420 (0.649, 2.191)	
Indirect effect	0.025 (-0.113, 0.195)	0.024 (-0.113, 0.213)	-
**LVEF**			
Total effect	-5.361 (-7.585, -3.137)	-5.764 (-8.606, -2.923)	
Direct effect	-4.924 (-7.208, -2.641)	-5.385 (-8.274, -2.495)	
Indirect effect	-0.437 (-1.161, 0.150)	-0.379 (-1.120, 0.235)	-
**E/A ratio**			
Total effect	-0.110 (-0.212, -0.007)	-0.096 (-0.222, 0.030)	
Direct effect	-0.076 (-0.181, 0.028)	-0.074 (-0.201, 0.054)	
Indirect effect	-0.033 (-0.071, 0.000)	-0.022 (-0.056, 0.013)	-
**Renal function**			

**BUN**			
Total effect	1.485 (0.846, 2.124)	1.588 (0.789, 2.386)	
Direct effect	1.413 (0.760, 2.066)	1.538 (0.727, 2.349)	
Indirect effect	0.072 (-0.115, 0.264)	0.050 (-0.136, 0.247)	-
**Creatinine**			
Total effect	30.331 (14.045, 46.618)	38.003 (17.780, 58.226)	
Direct effect	31.324 (14.651, 47.997)	39.177 (18.638, 59.716)	
Indirect effect	-0.993 (-6.973, 3.669)	-1.174 (-7.174, 3.771)	-
**CysC**			
Total effect	0.207 (0.071, 0.344)	0.247 (0.078, 0.416)	
Direct effect	0.211 (0.071, 0.351)	0.254 (0.083, 0.426)	
Indirect effect	-0.004 (-0.050, 0.040)	-0.007 (-0.056, 0.039)	-
**eGFR**			
Total effect	-13.434 (-20.000, -6.869)	-12.225 (-20.089, -4.422)	
Direct effect	-11.787 (-18.440, -5.133)	-11.172 (-19.093, -3.250)	
Indirect effect	-1.648 (-3.784, -0.099)	-1.084 (-3.227, 0.524)	-
**uACR**			
Total effect	178.743 (156.739, 200.747)	175.714 (148.433, 202.995)	
Direct effect	186.691 (164.651, 208.730)	182.722 (155.497, 209.947)	
Indirect effect	-7.948 (-14.641, -2.161)	-7.008 (-15.168, -1.054)	3.99%

[a]Adjusted for sex, age, BMI, smoke, alcohol, DM, hyperlipemia, antiplatelets, statin, ACEI/ARB, β-blocker, and SGLT2i. HTN, hypertension; EAT, epicardial adipose tissue; CACS, coronary artery calcification score; CT-FFR, computerized tomography-fractional flow reserve; RA, right atrium; LA, left atrium; RV, right ventricle; LVDd, left ventricular end-diastolic diameter; IVS, interventricular septum; LVPW, left ventricular posterior wall; PA, pulmonary artery; LVEF, left ventricular ejection fraction; BUN, blood urea nitrogen; CysC, cystatin C; eGFR, estimated glomerular filtration rate; uACR, urinary albumin-to-creatinine ratio; BMI, body mass index; DM, diabetes mellitus; ACEI, angiotensin converting enzyme inhibitor; ARB, angiotensin receptor blocker; SGLT2i, sodium-dependent glucose transporter 2 inhibitor.
